# The Effect of Flame Retardant—Aluminum Trihydroxide on Mixed Mode I/II Fracture Toughness of Epoxy Resin

**DOI:** 10.3390/polym14204386

**Published:** 2022-10-17

**Authors:** Paweł Zielonka, Szymon Duda, Grzegorz Lesiuk, Wojciech Błażejewski, Magdalena Wiśniewska, Joanna Warycha, Paweł Stabla, Michał Smolnicki, Bartosz Babiarczuk

**Affiliations:** Faculty of Mechanical Engineering, Wroclaw University of Science and Technology, PL50370 Wrocław, Poland

**Keywords:** fracture toughness, mixed mode, flame-retardants, epoxy resin, aluminum trihydroxide

## Abstract

Fire resistance is a major issue concerning composite materials for safe operation in many industrial sectors. The design process needs to meet safety requirements for buildings and vehicles, where the use of composites has increased. There are several solutions to increasing the flame resistance of polymeric materials, based on either chemical modification or physical additions to the material’s composition. Generally, the used flame retardants affect mechanical properties either in a positive or negative way. The presented research shows the influence of the mixed-mode behavior of epoxy resin. Fracture toughness tests on epoxy resin samples were carried out, to investigate the changes resulting from different inorganic filler contents of aluminum trihydroxide (ATH). Three-point bending and asymmetric four-point bending tests, with different loading modes, were performed, to check the fracture behavior in a complex state of loading. The results showed that the fracture toughness of mode I and mode II was reduced by over 50%, compared to neat resin. The experimental outcomes were compared with theoretical predictions, demonstrating that the crack initiation angle for higher values of KI/KII factor had a reasonable correlation with the MTS prediction. On the other hand, for small values of the factor K_I_/K_II_, the results of the crack initiation angle had significant divergences. Additionally, based on scanning electron microscopy images, the fracturing of the samples was presented.

## 1. Introduction

The safety issue is one of the most significant factors that any designed structure needs to consider while in service. Structural fire is one of the most dangerous threats that affects safety. Industry, supported by researchers, is continually developing advanced fire-resistant materials, which reduce fire risk and limit flame spread. In civil structures, all materials are classified according to the EN13501 standard [1], which determines the degree of flammability of the material and the release of gaseous poisonous combustion products. This requirements increases the safety of the users of the structure, provides sufficient time for evacuation, and meets the principles of fire prevention. As a result of compliance with the recommendations of the standards, there has been a decrease in fire deaths in recent years [2]. These highlighted problems of flammability mainly affect plastics and composite materials, which are additionally combustible [3].

Referring to composite materials, several manufacturing technologies can be highlighted, such as hand laminating, vacuum bagging [4], infusion [5], pultrusion [6,7], and filament winding [8,9,10,11]. Those processes allow manufacturers to provide the very complex material geometries and structures used in many modern applications. 

Nonflammable properties in many materials can be obtained by modification of the material structure using various fillers. Those modifications are essentially based on two methods: additive (non-reactive) and reactive [12]. Reactive-type methods rely on chemical modification of the polymeric structure, and additive-type flame retardants are based on physical addition to the material. Additive fillers can be divided into an organic and inorganic group [13,14,15,16]. The highlighted groups of additive fillers are characterized as follows:

- Flame-retardant organic compounds bound to the polymer during synthesis or cross-linking. Such compounds (mainly used for polyesters, polyurethanes, or epoxides) include, for example, pentabromophenol, and polyols containing phosphorus, nitrogen, or halogens.
- Compounds non-reactive towards the polymer are usually added during the processing of thermoplastics. This group includes, among others, halogen compounds, brominated diphenyl oxides, chlorinated and brominated paraffins, antimony compounds, aluminum and magnesium hydroxides, boron compounds, and melanin derivatives [17].

Additive fillers can be divided into organic and inorganic flame retardants [13,14,15,16]. Inorganic fillers are commonly used in industry, due to their price and due to environmental reasons. Regulations prohibit the use of halogen-containing flame retardants in polymeric materials because they are harmful to the environment [18,19]. Currently, the most popular method of flame retardation of plastics is using nanofillers containing aluminosilicates. These are environmentally friendly, unlike traditional composites with the addition of halogens or phosphates. Such nanocomposites emit less carbon monoxide and soot, which can be observed in the case of composites with traditional flame retardants [20]. The introduction of small amounts of filler improves various material properties, such as the tear strength, compressive strength, thermal expansion coefficient, and chemical resistance [21,22]. Very good nanocomposite fire properties result from the structure of these materials. Under the influence of high temperature and flames, a robust and charred layer without cracks is formed on the material’s surface, isolating the polymer from the fire, and this limits the access of flammable products of polymer decomposition to the combustion zone. Among aluminosilicates, the following can be mentioned:

- colloidal silica and kaolinite—require the use of high concentrations, which unfortunately deteriorates the mechanical properties of polymeric materials,
- montmorillonite—it is recommended to mix this with metal hydroxides, which improves the effectiveness of protection and allows the reduction of the concentration of flame retardants; a concentration of 5–10 phr is sufficient, which simultaneously improves the mechanical properties of polymeric materials,
- aluminum hydroxide Al(OH)_3_— used in a concentration even above 60 phr, forming a glassy layer on the surface of the polymer material under the influence of heating, which prevents fire propagation. During combustion, metal hydroxides decompose and release water. This decreases the temperature of the matrix, reduces the quantity of oxygen in the air, reduces the toxicity of exhaust gases, and causes endothermic dehydration, absorbing the heat [23]. One of the main disadvantages is the amount of filling required to obtain the desired flame-resistance effect, which can be up to 40 %wt. This can lead to a reduction of the fluidity of the resin and deterioration of mechanical properties. It also significantly influences industrial processing, such as mixing, molding, and fiber wettability. Magnesium hydroxide Mg(OH)_2_—in a concentration above 60 phr, this reduces the flammability of polymeric materials exposed to higher temperatures, where Al(OH)_3_ is not very stable. Unfortunately, it worsens the mechanical properties of the material.

Using silica particulate-filled epoxy composites, the fracture toughness K_IC_ increased with a higher quantity of filler [24]. In this material, with a constant radius of the silica particles, a relationship between the fracture toughness and glass transition temperature was observed. For pure resin, this dependence was not noticed. If the grain size was considered, the filler with a smaller particle diameter resulted in a more significant increase in the fracture toughness value [25].

Graphene nanosheets and carbon nanotubes have also been studied to improve the flame retardancy of polymers. The results showed that a small volume of graphene nanosheets could improve the LOI value [26]. A combination with phosphorus-containing flame retardants could increase the flame retardance properties of the composition, due to the use of the advantages of each component [27]. Considering environmental problems, works on biodegradable flame retardants are underway [28,29,30]. Materials such as chicken feathers, clamshells, or empty fruit bunches were considered. 

For construction materials, one of the most critical parameters is the mechanical properties. More detailed research on the influence of the filler on mechanical properties was conducted for materials exhibiting the flame retardance properties mentioned earlier. Tests of various types of fillers were carried out, determining the influence of the microstructure, chemical composition, and dispersion on the matrix properties [31,32,33]. It has been shown that materials with a more complex structure and no aggregation tendency can increase a resin’s mechanical properties. For instance, a mixture of carbon nano-fillers, graphene nanoplates, and multi-walled carbon nanotubes increased the interlaminar fracture toughness of Mode I and Mode II [34]. The results showed that these hybrid concepts had a greater effect on the improvement of the properties than the individual filler, with an equal amount of addition. The carbon nanotubes were also blended with thermoplastic particles, which significantly increased the fracture toughness of the epoxy matrix, due to their different energy absorption mechanisms [35]. The main disadvantage of carbon nano-fillers is their price, which could be prohibitive for many applications. As the main application of modified resin in the present study is in the matrix of reinforcement rebars made by the pultrusion process, and as price is a crucial factor, the preliminary investigation was focused on aluminum hydroxide Al(OH)_3_. Ensuring non-flammability is a necessary point to guarantee the safety of humans and constructions.

Research conducted on a PMMA matrix and epoxy matrix showed that the main impact of the fracture toughness came from the size of the particle and volume fraction [36,37]. The fracture toughness increased with a greater size of particles and for low filler contents. After exceeding the content limit, the value of the parameter decreased. As well as the particle size, another important aspect is the distribution of the particles in the matrix volume. If the filler tends to agglomerate, the strain concentration becomes higher, and this can easily lead to the critical stress criterion of debonding between the matrix and surface of the particle [37]. The simple arrangement of two particles loaded with unidirectional force was proposed by Evans to explain the debonding process of the particle [38]. Another modification of epoxy resin with an aluminum hydroxide flame retardant was the addition of phosphorus function and a mixture with antimony trioxide [39]. The results showed that the stablished values of the fillers, tensile strength, and Young’s modulus were greater than that of the initial mixture. The burning time was also increased in the UL-94 test. A fracture toughness test was not carried out. Aluminum hydroxide has also been used in other polymer matrixes, such as unsaturated polyester [40]. The best results were obtained for a 10% content of filler, where the tensile strength was higher than for pure resin but the fracture elongation was simultaneously decreased. Nano-aluminum hydroxide has also been used with thermoplastic polymers, such as polypropylene [41]. The addition of a mere 2% nano-ATH was capable of increasing the tensile strength and elongation to break compared to the primary material. It also reduced the mass loss time. An investigation was also conducted on samples made of epoxy resin with chopped strand glass fiber and three levels of ATH filler [42]. The authors noted that the flexural strength was reduced during the addition of the aluminum hydroxide filler and the material became more brittle. 

The properties of the matrix are crucial for composite structures to correctly transfer the load to the fiber reinforcement. Reducing the mechanical performance of the resin leads to under-utilization of the fiber capacity and premature destruction of the structure [43,44,45,46,47]. Fracture toughness tests could provide the resistance of the material to cracks, propagation in material, and sharp notches on the surface. The fracture mechanism of composite parts may occur in an interlaminar, intralaminar, or translaminar form [48,49,50,51,52]. Interlaminar failure develops between the layers of the structures, causing their separation.

During this research, the impact of mixed mode I and mode II with varying compilations to crack propagation was investigated. These parameters are not usually attached to the datasheet of resins, as is information about the tensile strength or Young’s modulus, but they are crucial to predicting the fracture behavior of the laminates. In this investigation, the fracture parameters of a thermoset resin were calculated based on linear elastic fracture mechanics (LEFM) [53]. Mechanical properties were investigated using three-point bending and fracture toughness tests. The analysis of the crack path was carried out, and the empirical results were compared with the theoretical predictions. In addition, the morphology of the nanocomposite and the fracture surface was characterized using scanning electron microscopy (SEM/EDX) and an optical microscope. 

## 2. Materials and Methods

### Materials

The resin system used in this research is a three-component, anhydride cured, thermosetting epoxy resin, Biresin CR141 from Sika Company. It is particularly suited to the filament winding and pultrusion process, due to its low viscosity and long pot life. The components’ mechanical properties and mixing ratio are presented in Table 1 and Table 2. More precise information about the resin composition is restricted by the company. 

Based on the literature review, a target group of flame retardants was selected [54,55,56,57]. The investigation concentrated on inorganic flame retardants, such as aluminum silicates (colloidal silica, kaolin, montmorillonite) and metal hydroxides (Al(OH)_3_ and Mg (OH)_3_), due to their excellent thermal stability, nontoxicity, and low price, which is essential for industrial applications. 

The samples were prepared using open mold casting. The material of the mold was soft and flexible with two-component silicone. The samples were rectangular-shaped with dimensions of 90 × 20 × 10 mm. To verify the resin’s mechanical properties, fracture toughness tests were carried out.

**Figure 1:**
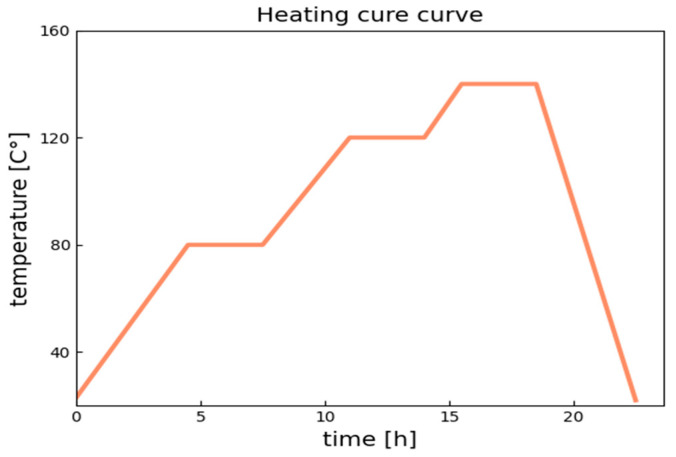
Heating cure curve used for curing resin samples.

The heating cure curve was plot based on the resin system’s product data-sheet. The temperature was held on for 3 h at three different levels: 80 °C, 120 °C, and 140 °C. The heat-up rate was 0.2 °C/min, and the resin was cooled at 0.5 °C/min. The heating curve is presented in Figure 1.

Samples for mechanical tests were prepared with different ATH filler (R&G Company) content ratios: pure resin, 30 phr Al(OH)_3_, and 50 phr Al(OH)_3_. The mean diameter of the particles was 1 µm. After demolding, the surface and geometry of samples were prepared with a rotary grinder. A cut was made using a diamond wire saw machine to a 9-mm depth. The front of the incision was sharpened by making several cuts with a scalpel. 

**Figure 2:**
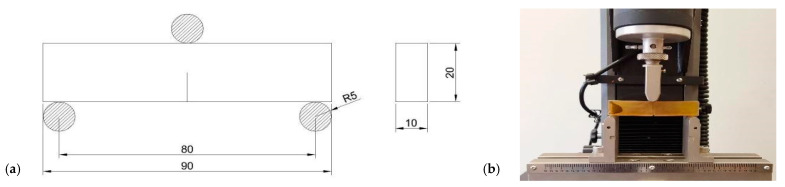
(**a**) Dimensions of the fracture toughness test sample (in mm). (**b**) Fracture toughness tests performed on the testing machine, 3-point bending test.

**Figure 3:**
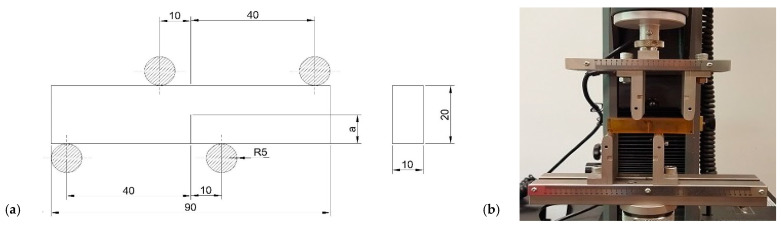
(**a**) Dimensions of the fracture toughness test sample (in mm). (**b**) Fracture toughness tests performed on the testing machine, 4-point bending test.

Based on previous studies of epoxy materials, comparing fracture toughness values depends on many parameters. One is the thermo-viscosity effect, which makes the results time-temperature dependent. Increasing the displacement rate of the loading point could significantly change the K_I,_ and K_II_ values. Mode II fracture is more sensitive to this phenomenon [58,59]. Tests were conducted using a universal material testing machine at room temperature with a constant displacement rate of 2$\frac{\mathrm{mm}}{\min}$. The machine was equipped with a static load cell with a 2 kN capacity. During the static tensile test, strains were measured using an external extensometer with a measuring base of 25 mm. After the test, Young’s modulus was calculated. The measurement stand and SENB (single edge notched bend specimen) specimen are presented in Figure 2 and Figure 3.

Suppose the shape of load–displacement curve is linear until the breaking of the specimens. In that case, the linear elastic fracture mechanics can be applied to determine the mixed mode I/II fracture toughness. This is caused by the small-scale yielding in the stress field near the crack tip. The stress intensity factor, *K*_I_ and *K*_II_, can be calculated by [60,61,62]:

(1) $$K_{I}=\sigma_{0}F_{I}\sqrt{\pi a}$$

(2) $$K_{\mathrm{II}}=\sigma_{0}F_{\mathrm{II}}\sqrt{\pi a}$$

The factors σ_0_, *F*_I_ and *F*_II_ were different for the 3-point bending test (3PBT) and asymmetric 4-point bending test (A4PBT). The equations to calculate the 3PBT factors are:

(3) $$\sigma_{0}=\frac{3PL}{W^{2}B}$$

(4) $$F_{I}=F_{I3}{\left(1-\frac{a}{W}\right)}^{-3/2}$$

(5) $$F_{\mathrm{II}}=F_{\mathrm{II}3}{\left(1-\frac{a}{W}\right)}^{-1/2}$$

The factors $F_{I3}$ and $F_{\mathrm{II}3}$ are geometric functions which describe the complexity of the stress state at the end of the crack tip for the tree-point bending test. The parameters such as crack length a, width W, thickness B, and distance between supports L represent the dimension of the sample and experimental setup. The load P is taken directly from the testing machine. The equations to calculate the A4PBT factors are:

(6) $$P=\frac{d}{L+d}p$$

(7) $$\sigma_{0}=\frac{P}{WB}$$

(8) $$F_{\mathrm{II}}=F_{I4}\;\left(1-\frac{d}{L}\right)$$

(9) $$F_{\mathrm{II}}=F_{\mathrm{II}4}\;\left(1-\frac{d}{L}\right)$$

In the experiment, an asymmetric four-point bending test was conducted. For the three-point bending tests, $F_{I4},F_{\mathrm{II}4}$ are geometric functions depending on the geometrical conditions of the sample. The used dimensions of the sample and setup were the distance between the center of a sample and central support d, distance between supports L, width W and thickness B of the sample. The main difference compared to 3PBT was the determination of the applied load. In A4PBT, the applied load was calculated using the load from the testing machine and a factor based on two parameters: the distance between supports L and the distance between the center of the sample and central support d. The factors $F_{I3},F_{\mathrm{II}3},F_{I4},F_{\mathrm{II}4}$ were taken from [60,61].

## 3. Results and Discussion

In mixed-mode fatigue crack propagation, an essential part of the investigation is the analysis of the crack paths; the angular direction (crack branching) of crack growth (*ψ*_0_). The most widely used criteria for mixed-mode crack propagation are the maximum energy release rate (MERR), maximum tangential stress (MTS), and strain energy density (SED). The most widely used criterion in fracture mechanics is the MTS criterion and its generalizations [63]. The MTS criterion assumes that the direction of initial crack growth follows this direction, where the tangential stress reaches its maximum value. For the generalization of mixed-mode loading conditions, the elastic–mixity parameter *M_e_* can be defined as:

(10) $$M_{e}=\frac{2}{\pi }arctan\left(\frac{K_{I}}{K_{\mathrm{II}}}\right)$$

The value $M_{e}=0\;$corresponds to a pure shear state (mode II) and the value *M_e_* = 1 means that the specimen is loaded in pure mode I. Knowing the values of *K*_I_ and *K*_II_, the initiation angle in the MTS criterion can be determined, as follows:

(11) $$\psi_{0}=\left(\frac{3K_{\mathrm{II}}^{2}+\sqrt{K_{I}^{4}+8K_{I}^{2}K_{\mathrm{II}}^{2}}}{K_{I}^{2}+9K_{\mathrm{II}}^{2}}\right)$$

**Figure 4:**
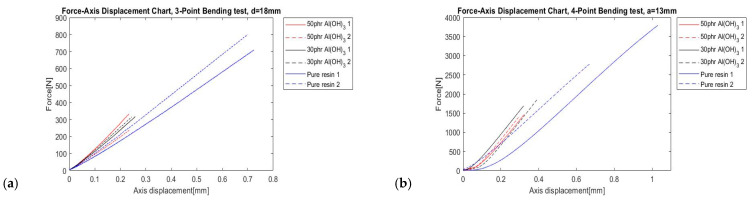
Reference load–displacement curve at the loading point (**a**) 3PBT (**b**) A4PBT.

The angular direction of crack growth could also be estimated from the experimental tests. Three-point bending and four-point bending tests were carried out, and reference results from one set each with 3PBT and A4PBT are presented in Figure 4.

Considering the results from Figure 4, a significant decrease in maximum force is noticeable, which may suggest negative an influence of the filler. Additionally, the curve slopes vary, which was caused by the slightly different sample thickness. Furthermore, the results from the experimental part exhibit the influence of mode II on the maximum force. Mode I affected the investigated samples more, significantly decreasing the maximum force. Increasing the mode-mixity with mode II (in-plane shear) the maximum force increased up to 3000 N. 

**Figure 5:**
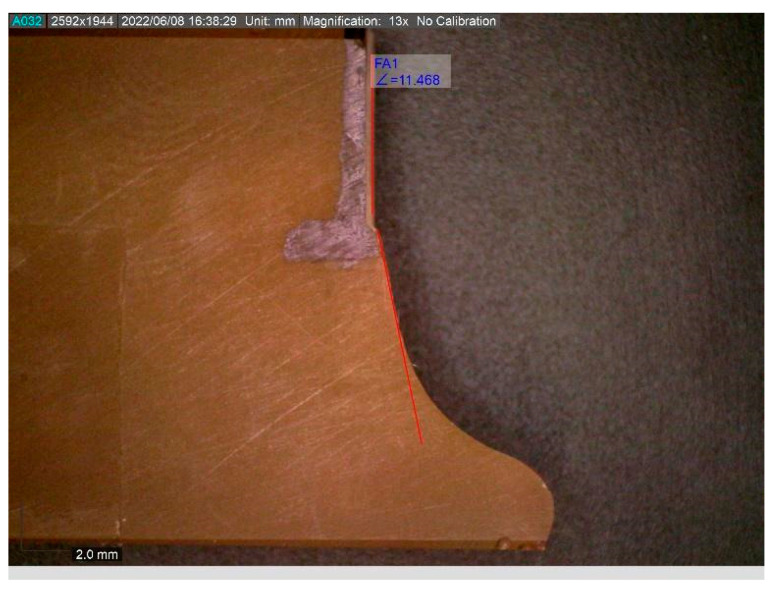
Estimation of angular direction of crack growth using a digital microscope.

After mechanical tests, the angle between the incision and crack growth was checked using a digital microscope, Dino-Lite. The calculation of the angle is shown in Figure 5. The measured values were compared with the prediction, to evaluate the correlation between experimental and analytical approaches. 

**Figure 6:**
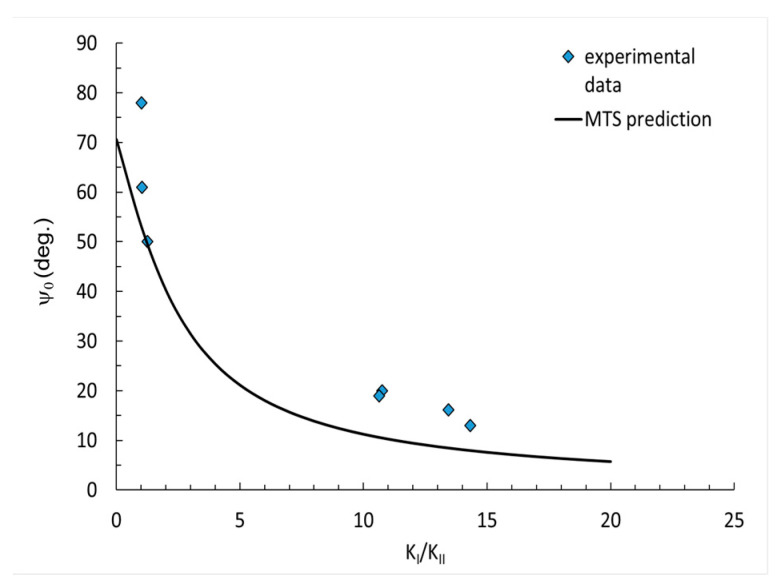
Crack initiation angles for pure resin.

**Figure 7:**
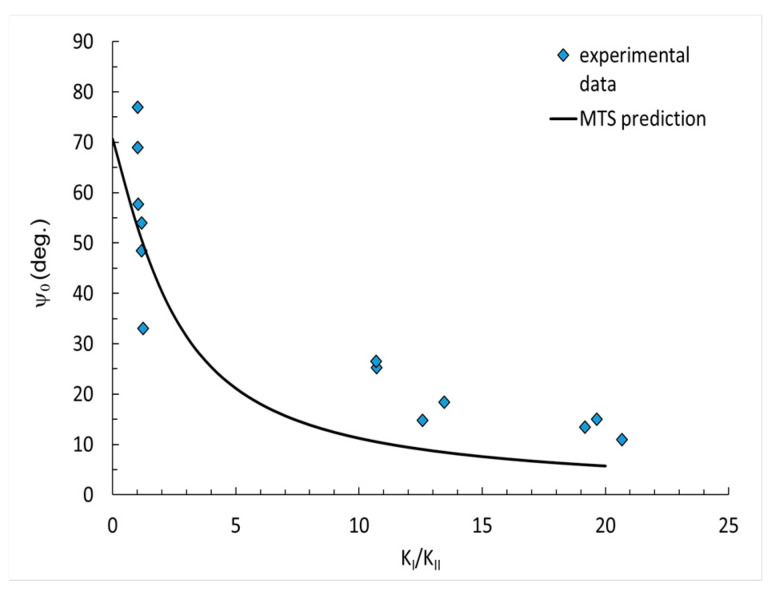
Crack initiation angles for 30phr Al(OH)_3_.

**Figure 8:**
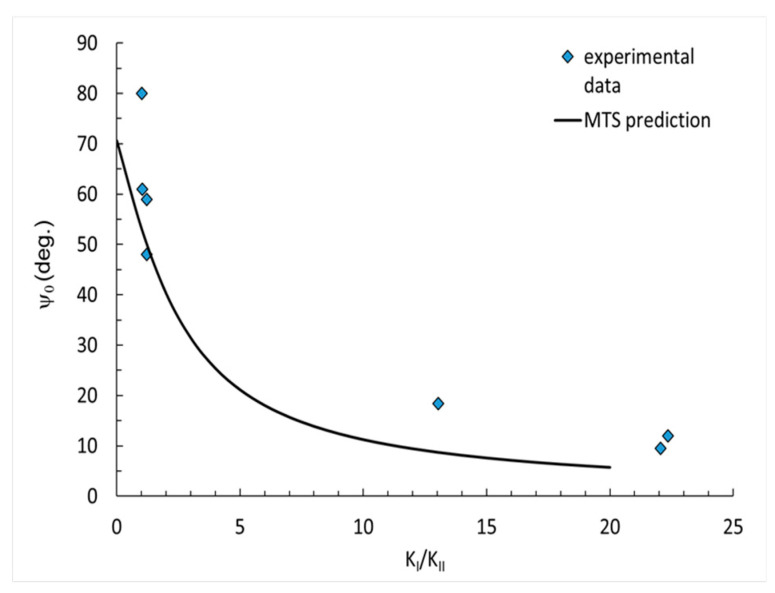
Crack initiation angles for 50 phr Al(OH)_3_.

The results from the MTS prediction and experimental data were compared and are presented in Figure 6, Figure 7 and Figure 8. The results are divided into three diagrams with different contents of flame retardant: pure resin, 30 phr Al(OH)_3_, 50 phr Al(OH)_3_. The measured crack initiation angle was larger than that in the MTS prediction. The data best fit the small values of K_I_/K_II_ factor.

The applied MTS prediction for the fracture toughness ratio did not reflect the correct values, specifically for smaller angels. Basically, the experimental data (blue points) was ordered in a similar manner as the predicted curve, which may suggest that some correction factor for the MTS prediction might be useful and increase the accuracy. 

**Figure 9:**
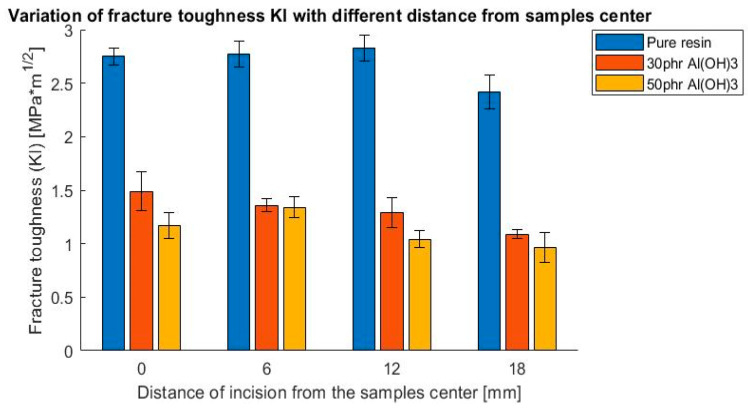
Variation of fracture toughness K_I_ with different distances from sample center, 3-point bending test.

**Figure 10:**
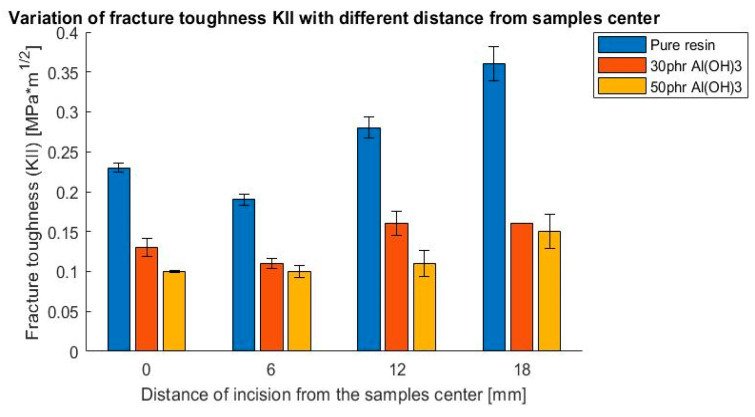
Variation of fracture toughness K_II_ with different distances from the sample center, 3-point bending test.

**Figure 11:**
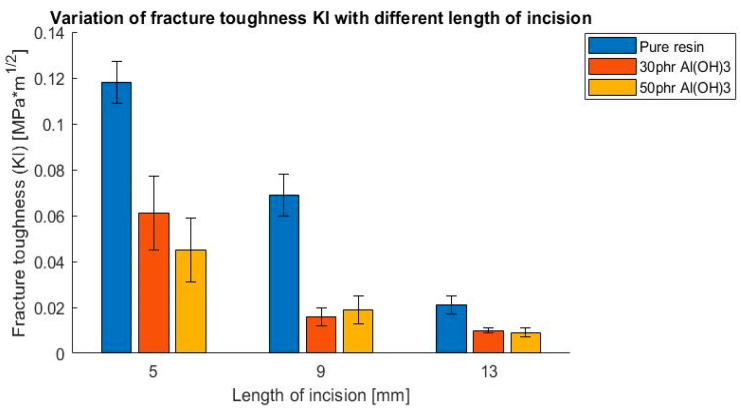
Variation of fracture toughness K_I_ with different lengths of incision, 4-point bending test.

**Figure 12:**
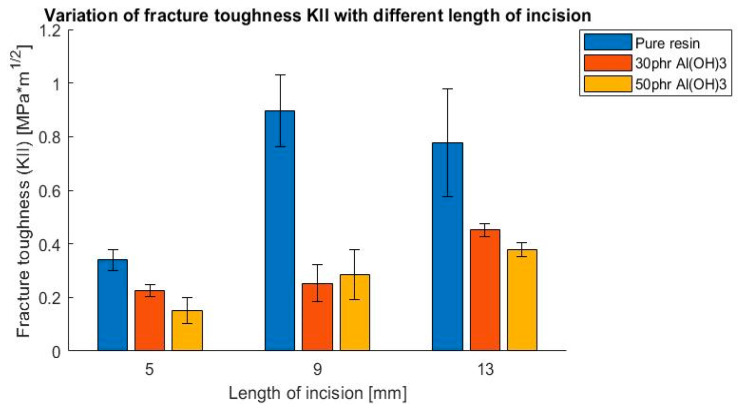
Variation of fracture toughness K_II_ with different lengths of incision, 4-point bending test.

As a result of these tests, the influence of filler addition on the values of the fracture toughness coefficient K_I_ and K_II_ were determined. The outcomes are presented in Figure 9, Figure 10, Figure 11 and Figure 12, presented as a bar chart grouped using the mode I and II ratio. Considering the presented results, it might be indicated that the addition of flame retardant significantly impacted the fracture toughness properties, decreasing the values by roughly 50%. Additionally, it is noticeable that the differences between the flame retardant contents did not cause notable changes in the results. This suggests that basically the fracture behavior of the epoxy resin was affected by the fillers decreasing the cohesive force, independently of the volume content. 

**Figure 13:**
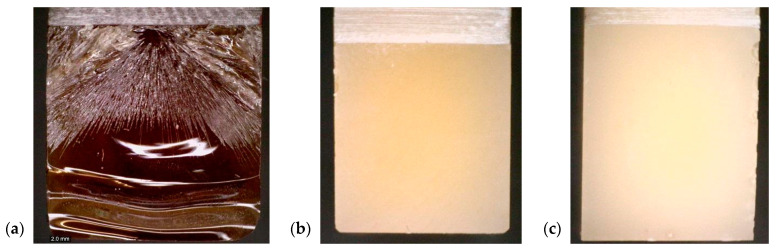
The fracture surface of samples after fracture toughness tests: (**a**) neat resin (**b**) 30 phr ATH (**c**) 50 phr ATH.

An exploratory inquiry based on the material microstructure was conducted using microscopy. Several tests were performed, to determine the dispersion of the additive in the structure, the nature of the cross-section, and the true filler content of the material. In Figure 13 the impact of a flame retardant on a cross-section surface is presented using an optical microscope DinoLite. The most significant differences can be noted at the point of fracture initiation. The surface has numerous cracks and lines, indicating crack propagation. At the end of the sample, the surfaces become wavy and smooth. The addition of ATH filler increased the brittle nature of the fracture of the material. The cross-section surface was homogeneous, without any changes to the surface’s beginning and end.

A series of SEM photos were taken to study the form of propagation in the material. The images also exhibited a relatively smooth fracture surface, with a cracks in different planes. The cracks progressed along from the place where the fracture was initiated. The numerous cracks on the surface structure after failure may explain the much higher value of the fracture toughness coefficient for pure resin. The fracture surface of pure resin was typical for such materials and was composed of zones such as “mirror” surface, mist, and hackle. The hackle surface is shown in Figure 13a. 

**Figure 14:**
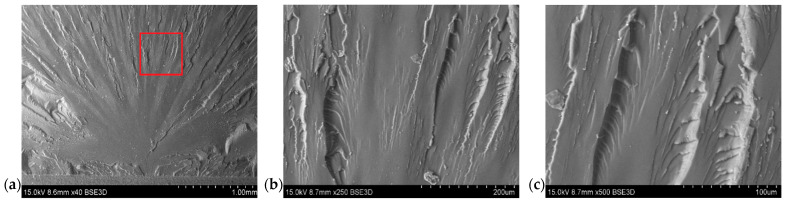
Fracture surface of pure resin material in SEM images. (**a**) Crack initiation zone with the marked area with further enlargement. (**b**) Fracture surface of neat resin-magnification ×250. (**c**) Fracture surface of neat resin-magnification ×500.

**Figure 15:**
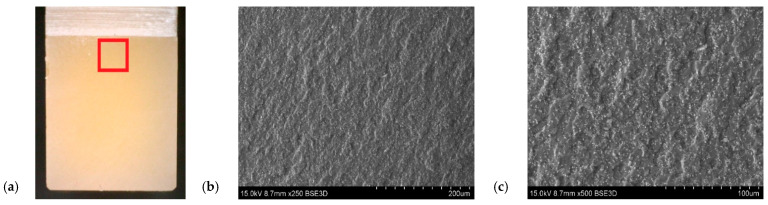
Fracture surface of resin with the addition of a filler 30 phr Al(OH)_3_, white points representing ATH filler. (**a**) Sample with marked SEM images location. (**b**) Fracture surface with magnification ×250. (**c**) Fracture surface with magnification ×500.

**Figure 16:**
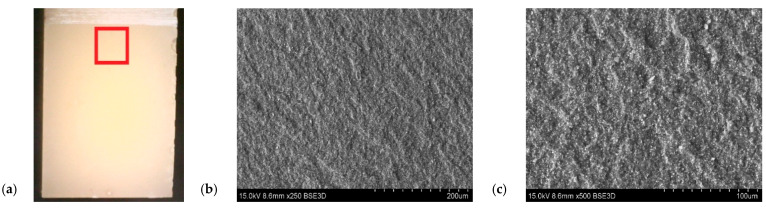
The fracture surface of resin with the addition of a filler 50 phr Al(OH)_3_. (**a**) Sample with marked SEM images location. (**b**) Fracture surface with magnification ×250. (**c**) Fracture surface with magnification ×500.

The filler dispersion in the resin structure was also taken into account, the content of which was determined using SEM microscopy. The particles of flame retardant were well dispersed in the sample volume. Figure 14 show a significant visual difference in the filler content between the samples. On the other hand, Figure 15 and Figure 16 show fractograms from specimens with flame-retardant particles. It is worth noting that the fracture mechanism significantly differed from pure resin materials. Notably, there are separated particles in the “hackle” region. The SEM images in Figure 14, Figure 15 and Figure 16 indicate that the applied filler increased the fracture surface roughness. The increased surface roughness implies that the path of the crack tip was distorted because of the flame retardant particles, making the crack propagation more demanding. This corresponds with the SEM images of the resin with different contents of filler, with the lines indicating the crack propagation being more deformed in the resin with a higher content of filler. This means that the crack tip was more deflected and meandering in the matrix. In both cases it was impossible to designate the crack initiation. Owing to the specific size of the particles, the crack tip interacted with the filler and changed the crack trajectory. However, the size was insufficient to increase the fracture toughness parameter, due to extended the crack tip propagation. Due to the huge amount of particles in the resin volume and their good distribution, the crack tip moved between grain boundaries, where the stress concentration occurred. This phenomenon may have been responsible for the decreased fracture toughness.

**Figure 17:**
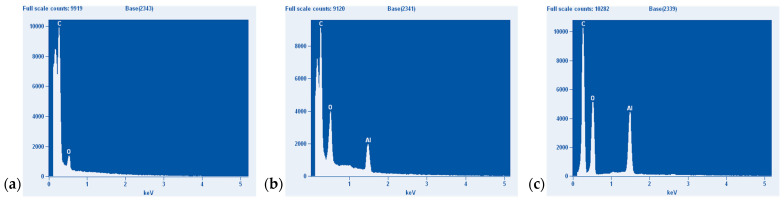
Results from the EDX analysis: (**a**) neat resin, (**b**) resin with 30 phr ATH, (**c**) resin with 50 phr ATH.

More precise data on the content of elements were obtained from the EDX analysis presented in Figure 17 and Table 3. The analysis confirmed the higher content of filler in the 50 phr ATH sample. 

## 4. Conclusions

In the paper, the mixed mode I/II fracture behavior of epoxy resin at room temperature was investigated. Mechanical tests, such as a 3-point and 4-point bending tests with different mode-mixity were conducted, showing the influence of the applied filler content on the fracture toughness. Based on the research performed, the following conclusions can be drawn:

- The addition of ATH filler, despite the increase in flame resistance, causes a significant decrease in fracture toughness resistance. The fracture toughness value was reduced by more than 50%, when comparing neat resin with the modified resin.
- Changes in fracture toughness between the resin with 30 phr Al(OH)_3_ and 50 phr Al(OH)_3_ filler were insignificant.
- The comparison of the experimental data and analytical prediction showed that the experimental data of the crack initiation angle for higher values of K_I_/K_II_ factor had a reasonable correlation with MTS prediction.
- For small values of the factor K_I_/K_II_, the results of the crack initiation angle had significant divergences. The points for the crack initiation angle measured after the experiment basically demonstrated a similar nature of curve as those obtained from the prediction. As such, it may be necessary to introduce a correction parameter, to better correlate with the MTS prediction.

The above conclusions provide an overview of the mixed-mode fracture behavior of epoxy resin, including the influence of ATH filler and a comparison of the experimental and analytical methods for predicting the crack initiation angle. This information was helpful during the designed process, as well as on the technological stage, for both the material and structure, and to provide the required operating conditions. However, the conducted research showed that a more complex investigation needs to be performed, which will provide more data on the flame resistance and ductility of the material.

## Figures and Tables

**Table 1:** Resin components.

	Biresin CR141 Sika Company	Biresin CH141 Sika Company	Biresin CA141 Sika Company
	Resin (A)	Hardener (B)	Accelerator (C)
Components	≥90% bisphenol-A-(epichloryhydrin) epoxy resin (number average molecular weight ≤ 700)	≥50%–≤100% tetrahydromethylphthalic anhydride	≥50%–≤100% 1-methylimidazole
Mixing ratio, parts by weights	100	90	2

**Table 2:** Mechanical data from the technical datasheet, properties after 3 h/80 °C + 3 h/120 °C + 3 h/140 °C.

Type of Properties	Standard	Value	
Tensile strength	ISO 527	78	MPa
Tensile E-modulus	ISO 527	3.2	MPa
Elongation at break	ISO 527	3.3	%
Flexural strength	ISO 178	145	MPa
Flexural E-Modulus	ISO 178	3.1	MPa
Density	ISO 1183	1.2	g/cm^3^

**Table 3:** Content of carbon, oxygen, and aluminum elements in the sample volume.

Element (wt%)	Pure Resin	30 phr Al(OH)_3_	50 phr Al(OH)_3_
C	79.54	48.56	50.54
O	20.46	45.59	41.78
Al	0	5.84	7.68

## Data Availability

The data presented in this study are available on request from the corresponding author.
